# Analysis of the Changes in Expression Levels of Sialic Acid on Influenza-Virus-Infected Cells Using Lectin-Tagged Polymeric Nanoparticles

**DOI:** 10.3389/fmicb.2016.01147

**Published:** 2016-07-21

**Authors:** Jaebum Cho, Yukari Miyake, Ayae Honda, Keiichiro Kushiro, Madoka Takai

**Affiliations:** ^1^Department of Bioengineering, The University of TokyoTokyo, Japan; ^2^Department of Frontier Bioscience, Hosei UniversityTokyo, Japan

**Keywords:** nanoparticles, sialic acid, lectin, polymers, infection

## Abstract

Viral infections affect millions around the world, sometimes leading to severe consequences or even epidemics. Understanding the molecular dynamics during viral infections would provide crucial information for preventing or stopping the progress of infections. However, the current methods often involve the disruption of the infected cells or expensive and time-consuming procedures. In this study, fluorescent polymeric nanoparticles were fabricated and used as bioimaging nanoprobes that can monitor the progression of influenza viral infection through the changes in the expression levels of sialic acids expressed on the cell membrane. The nanoparticles were composed of a biocompatible monomer to prevent non-specific interactions, a hydrophobic monomer to form the core, a fluorescent monomer, and a protein-binding monomer to conjugate lectin, which binds sialic acids. It was shown that these lectin-tagged nanoparticles that specifically target sialic acids could track the changes in the expression levels of sialic acids caused by influenza viral infections in human lung epithelial cells. There was a sudden drop in the levels of sialic acid at the initial onset of virus infection (*t* = 0~1 h) and at approximately 4~5 h post-infection. The latter drop correlated with the production of viral proteins that was confirmed using traditional techniques. Thus, the accuracy, the rapidity and the efficacy of the nanoprobes were demonstrated. Such molecular bioimaging tools, which allow easy-handling and *in situ* monitoring, would be useful to directly observe and decipher the viral infection mechanisms.

## Introduction

Recently, viral infection has been a major global issue due to the dangers associated with the high death ratio and secondary diseases (Sun et al., 2011; Chen et al., 2012; Sichelstiel et al., 2014), also causing chronic infections in the case of negligence (Landford et al., 2010; Jacobson et al., 2011; Chen et al., 2013). However, the molecular interactions and mechanisms behind these viral infections have not been clarified. Many types of analytical methods have been developed for viral infection such as polymerase chain reaction (PCR) and enzyme-linked immunosorbent assay (ELISA), but these assays involve disruptive cell lysis processes with expensive reagents due to the complicated nature of these protocols. In order to enable a detailed analysis and develop a deeper understanding of the viral infection process, it is necessary to develop a novel analytical system compatible with living cells.

During viral infection of cells, sialic acids on the cell membrane, which are oligosaccharides with glycoproteins and glycolipids, play essential roles. The influenza viral infection proceeds through the attachment of hemagglutinins (HA) on the viral membrane to sialic acids (de Lima et al., 1995; Fukuzawa et al., 2011; Lai et al., 2012; Zhu et al., 2012). HA is a viral coat glycoprotein, which binds to specific sialic acid receptors in the respiratory tract for penetration into the cell (Connor et al., 1994; Kuiken et al., 2006; Maines et al., 2009). In particular, specific kinds of influenza virus are associated with specific sialic acid binding and modifications for their pathogenic pathway, as well as the types of HA. When the influenza viruses penetrate into the cells for the infection, HA of the virus recognizes and binds to sialic acid on the cell membrane, and so, the expression level of sialic acid on the host cell is affected by viral infection (Ueda et al., 2013). Lectin, a class of carbohydrate-binding proteins, has high specificity for the specific sialic acid bound with other biomolecules. For example, *Sambucus nigra barks* lectin (SNA) can specifically recognize α-2,6-sialic acid and this type of sialic acid is known to be part of a recognizable glycan for binding by HA on viruses. To analyze the receptor-binding preferences, recognition mechanisms and changes in the expression levels of reacted sialic acids, it is necessary to assess the *in situ* changes in the cell membrane structures, such as glycans.

Recently, there have been multiple advances in bioimaging techniques to monitor cells using fluorescent nanoparticles, and they offer multiple advantages such as the capacity for real-time, non-disruptive monitoring of individual cells and ease of handling (Goto et al., 2008; Wolfbeis, 2015). In particular, a polymeric-nanoparticle-based bioimaging platform that can specifically and sensitively measure sialic acid levels have been developed (Cho et al., 2014). More specifically, this biocompatible bioimaging nanoprobe consist of 2-methacryloyloxyethyl phosphorylcholine (MPC), *n*-butyl methacrylate (BMA), *o*-nitrophenyloxycarbonyl polyethleneglycol methacrylate (MEONP), *p*-methacyloxyethyl thiocarbonyl rhodamine B (MTR), and lectin. MPC polymers prevent protein adsorption and thus makes the nanoprobe bioinert to most biomolecules (Moro et al., 2004; Goda et al., 2009). BMA is highly hydrophobic and thus, in conjunction with polylactic acid (PLA) forms the hydrophobic core of the nanoparticle. MEONP contains active ester groups that can conjugate other biomolecules, which in this case is streptavidin that subsequently bind the biotinylated lectin. MTR contains Rhodamine B, which have been safely used for the detection of mitochondria in the living cell in other bioimaging probes (Johnson et al., 1980). In this study, using these lectin-conjugated fluorescent polymeric nanoparticles as nanoprobes, simple and rapid analysis of sialic acid on the cell membrane were performed by tracking the fluorescence of the nanoprobes on the cells. This nanoprobe system was compared to some of the traditional methods to detect sialic acids, and it was suggested that these nanoprobes can be applied as a quick and reliable *in situ* bioimaging technology for the early detection of the changes in sialic acids and to further understand the process of viral infections.

## Materials and methods

### Reagents

Biotinylated *Sambucus nigra barks* lectin was purchased from Vector Laboratory (Burlingame, U.S.A). Phenylmethylsulfonyl fluoride (PMSF) was purchased from Calbiochem (Darmstadt, Germany). Anti-PB1 antibody was prepared by immunization of rabbit with purified PB1 protein. 4′,6-diamino-2-phenylindole (DAPI), rabbit immunoglobulin G (IgG) antibody and mouse IgG antibody with Alexa Flour 488 were purchased from Invitrogen (Carlsbad, CA, U.S.A). Ethylenediamine-N,N,N′,N′-tetraacetic acid (EDTA), N-cyclohexyl-3-aminopropanesulfonic acid (CAPS) and HEPES buffer solution were purchased from Dojindo (Kumamoto, Japan). Blocking one solution and Tris(hydromethyl) amino methane (Tris-base) were purchased from Nacalai tesque (Kyoto, Japan). Acrylamide, N′,N′-methylene bis(acrylamide) (bis), ammonium persulfate (APS), sodium dodecyl sulfate (SDS), paraformaldehyde (PFA) were purchased from Wako Pure Chemical Industries Co., Ltd. (Osaka, Japan). The details of the other reagents used for the nanoprobe fabrication and the cell probing are described in the previous study (Cho et al., 2014).

### Fabrication of bioimaging nanoprobes

The details of the fabrication process of the nanoprobes, as well as their chemical and physical characterizations, are described in the previous study (Cho et al., 2014). Briefly, the polymer base of the nanoprobe was synthesized via free radical polymerization by combining MPC, BMA, MEONP, and MTR monomers with α-2,2′-Azobisisobutyronitrile (AIBN), dissolved in degassed ethanol at 0.5 M for the monomers and 10 mM for AIBN. The solution was reacted in the oil bath at 65°C for 15 h then precipitated in the 8:2 (v/v ratio) mixture of diethylether and chloroform, respectively.

For the formation of the nanoparticles, the solvent evaporation technique (Goda et al., 2009) was utilized and lectin was subsequently conjugated. Briefly, 0.1 wt% of PLA in dichloromethane solution was mixed with 0.1 wt% of the aqueous polymer solution, and under the stirring condition at 400 rpm and 0°C, the mixture was sonicated using a probe-type sonicator (VP-5S, TAITEC, Japan) for 5 min. The formed nanoparticles were collected by an ultracentrifuge (XL-A, Beckman Coulter, U.S.A.) ran at 50,000 rpm at 4°C for 2 h, and lastly dispersed in deionized water. For the immobilization of lectins onto the nanoparticle surfaces to complete the nanoprobes, the nanoparticles at 10 mg/mL were first reacted with streptavidin at 10 μg/mL for 3 h, which in turn was conjugated with 10 μg/mL biotinylated SNA lectin for 3 h. In each reaction step, the products were collected by centrifugation at 50,000 rpm at 4°C for 2 h. It should also be noted that the modular design of the nanoprobe enables conjugation of different bioactive molecules to MEONP for various applications.

### Cell culture and viral infection

For cell culturing of human lung epithelial cell (H292 from American Type Culture Collection), the E-MEM, and EDTA solution were prepared. 9.8 g of E-MEM was added in 900 mL of deionized water. The solution was autoclaved for sterilization. After cooling of the autoclaved solution at the room temperature, FBS, L-glutamine and 7.5% of NaHCO_3_ were added in the solution. 7.5% of NaHCO_3_ was filtered with 0.2 μm of filter. In the serum-free E-MEM, other components except FBS were added. Two types of EDTA solution were prepared: 0.4% in water and 0.04% in DPBS.

To infect influenza virus into the cells, 3 × 10^4^ cells/well of H292 cells were cultured in the glass-bottom 96 well overnight. The cultured cells were washed with serum-free E-MEM medium. The washed cells were pre-incubated in 50 μL of the viral solution (multiplicity of infection of 1) for 1 h at 34°C. The solution included PR8 influenza A virus (H1N1) strain in the serum-free E-MEM. After pre-incubation, 100 μL of E-MEM medium was added in the dish. In the medium, the pre-incubated cells were cultured for infection at 34°C from 1 to 5 h.

### Western blotting and dot blot assay for detecting the expression level of sialic acid

For the Western blotting on the PVDF membrane, the cell proteins were extracted by cell lysis. Both the virus-infected and uninfected cells were cultured and sonicated five times for 10 s after addition of 0.1 M phenylmethanesulfonyl fluoride with the extraction buffer. The proteins of the sonicated cells in the solution were separated by centrifugation (15,000 rpm, 4°C, 30 min). The collected cell proteins were heated at 95°C for 3 min, and then the electrophoresis was performed. The samples including cellular proteins were separated on the SDS-PAGE at 30 mA for 1 h and soaked in 10 mM CAPS buffer containing 10% methanol (pH 11) for 20 min. The concentration of CAPS buffer (pH 11) was 0.01 M in deionized water (60-fold diluted from the original solution). After treatment with the membrane in CAPS buffer for 20 min, the sample was blotted by the electrophoresis on PVDF membrane (Nippon Genetics) at 4°C for 1 h. Then, the membrane was treated in the blocking one solution at 37°C for 30 min. On the membrane with cell proteins for various cell concentrations, 10 μg/mL of biotinylated SNA lectin were reacted for 12 h at the room temperature. After washing with DPBS twice in the rotary shaker (NA-301, Nissin, Tokyo, Japan) for 10 min, 10 μg/mL of Alexa-Fluor-488-conjugated streptavidin was reacted on the lectin-immobilized membrane for 12 h at the room temperature. The fluorescence images of the reacted membrane was washed with DPBS twice in the shaker for 10 min, and observed by image analyzer (Typhoon9410, GE Healthcare Life Sciences, Buckinghamshire, U.K.).

For the dot blot assay, attached cells in the dish were removed by treatment with trypsin. Removed cells were harvested by centrifugation at 700 rpm for 5 min at 4°C. The cell pellet was disrupted by adding glass beads in 100 μL Milli-Q water and centrifuged at 6500 rpm using Precelly 24 (Bertin, Provence, France) for 20 s, and placed on ice for 5 s. The disruption of cells was completed after 7 cycles. After the disruption of the cells, the supernatant was harvested by centrifugation at 15000 rpm for 5 min at 4°C and treated with trypsin adjusted to 0.01% for 30 min at 37°C. The supernatant equivalent to 10^5^ cells was dotted on the PVDF membrane, reacted with lectin and detected with the chemiluminescence using the luminescent image analyzer.

### Bioimaging of the expression level of sialic acid on the virus-infected cells using nanoprobes

Before the cellular experiment, H292 cells infected with influenza virus were incubated from 1 to 5 h at 34°C in the glass bottom 96-well plate with 100 μL of E-MEM medium. On the cells in the well plate, 10 μL of nanoprobe solution were added and reacted with H292 for 15 min at 34°C, respectively. The cells were then washed with PBS three times at room temperature, and then observed at 100x magnification using a fluorescence microscope (Nikon TiE microscope, Japan). The fluorescence intensities were quantified to determine the levels of sialic acid expression levels on the infected cells.

For the fixation of the cells to check HA expression, the influenza virus infected cells were washed twice with PBS at room temperature and fixed with 4% paraformaldehyde (PFA) for 15 min. After fixation, the cells were washed three times with PBS followed by reaction with anti-HA IgG (mouse) solution at 37°C for 1h. The cells were then washed with PBS three times at room temperature, reacted with anti-mouse IgG (rabbit) conjugated with Alexa 488 for 1 h at 37°C, observed at 100x magnification using the fluorescence microscope.

### Immunostaining for the confirmation of viral protein production

To demonstrate viral infection on the cells, immunostaining was performed. The virus-infected cells for various infection times were fixed using 100 μL of 4% paraformaldehyde in DPBS at room temperature for 15 min. Then, 100 μL of 0.1% of Triton X-100, a kind of detergent, in DPBS was reacted to permeabilize virus-infected cells for 5 min. After the reaction, the cells were incubated with 100 μL of 3% bovine serum albumin (BSA) in DPBS at the room temperature for 30 min. Then, the cells were reacted with 50 μL of 3% BSA solution including PB1/HA protein antibody at room temperature for 1 h. To perform fluorescent assay, the washed cells were reacted with Alexa-Fluor-488-conjugated rabbit IgG secondary antibody in 50 μL of 3% BSA solution at room temperature for 1 h. DAPI staining for the cell nucleus was also performed prior to the observation with the microscope. The reacted cells were washed five times with 100 μL of DPBS between each steps. The antibody-conjugated cells were observed using the fluorescence microscope.

### Real time polymerase chain reaction (RT-PCR) for the confirmation of transcription of viral protein genes

Total RNA was extracted from 10^5^ influenza virus-infected cells at each time point post-influenza virus infection using Qiagen RNeasy kit (Qiagen, USA). Primer sequences for RT-PCR were designed for the RNA 2 segment, which encodes PB1, a critical subunit of viral RNA polymerase. Forward primer was 5′-ACCGGAGACCCTCCTTACAGCC, reverse primer was 5′- TCGGGTTGAGTTGCGGTGCT. Then RT-PCR assay was performed using primers for RNA 2 segment. The emission from SYBR Green, a nucleic acid stain, was detected using an Applied Biosystems 7500 Real Time PCR System (Applied Biosystems, USA).

## Results

### Western blotting to identify the detection levels of α-2,6-sialic acid using lectin-fluorescence

**Figure 1:**
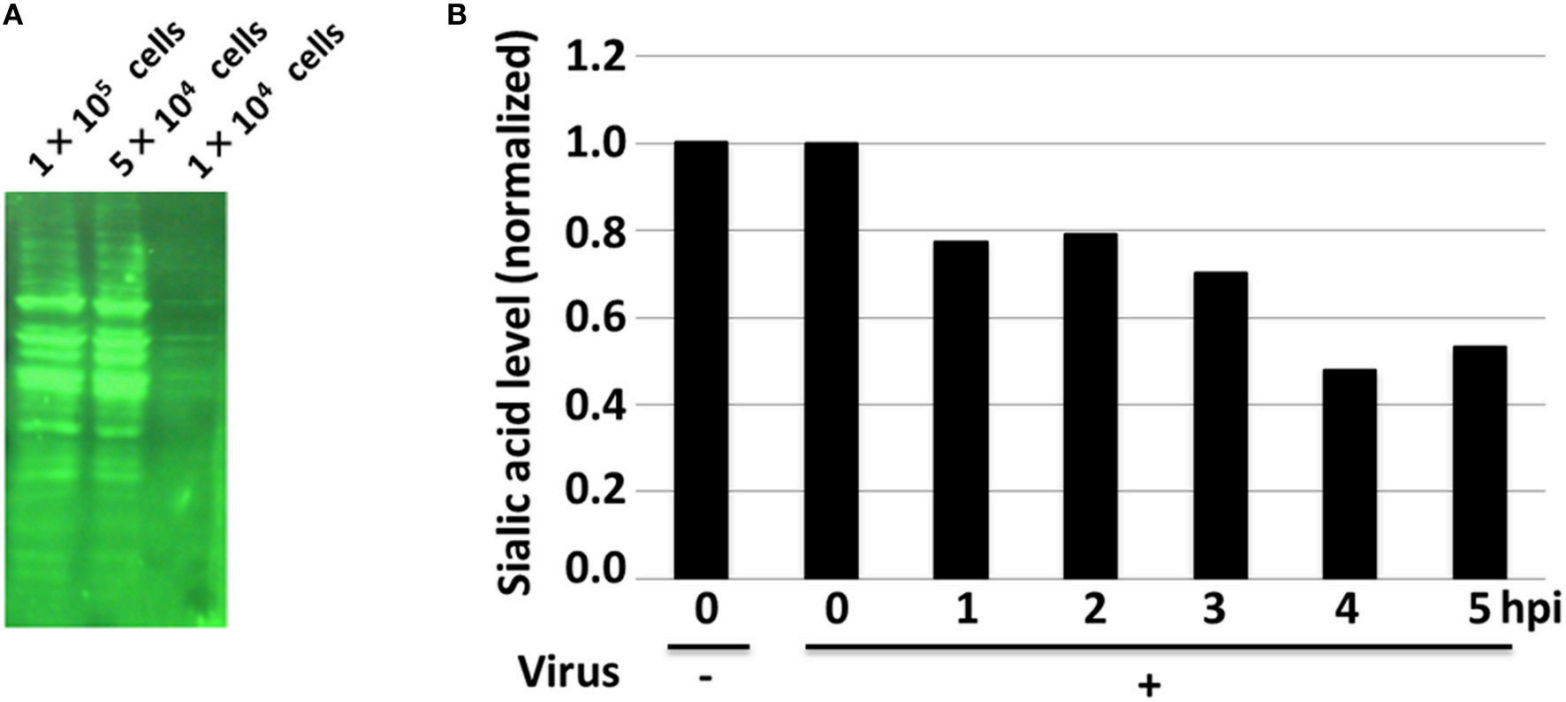
**The Western blot analyses of extracted α-2,6-sialic acids visualized using tagged-SNA lectins**. **(A)** Western blotting with different numbers of cells was performed to determine the optimal, non-saturating signals. **(B)** The quantification of the normalized chemiluminescence signals of the sialic acids are shown. Result for the control without viral infection (–) is also shown and the data set was normalized to the control.

Prior to the nanoprobe experiments on living cells, the presence and the relative amount of α-2,6- sialic acids in the H292 cells were examined using the traditional Western blotting approach. The blot was visualized using fluorescently-tagged streptavidin to perform Western blotting (Figure 1A). It was shown that the different concentrations of cellular proteins were transferred onto the PVDF membrane, and the signal intensity was confirmed to increase in proportion to the cell number. Below 1 × 10^4^ cells, the fluorescence intensity was sharply decreased, while above 5 × 10^4^ cells, the signal seemed to be saturated. Thus, for the subsequent Western blotting analysis, the optimal range of cell number to investigate the levels of sialic acid of H292 cells using lectin was calibrated to be between these numbers.

Next, the dot blot assay was performed using 2 × 10^4^ cells/dot to determine the relative amounts of sialic acid in the H292 cells at different time points after viral infection (Figure S1). The result showed that the amount of sialic acid in cells decreased in a step-wise fashion over time as the viral infection progressed, ultimately falling below 50% compared to the non-infected cells at 4 h post-infection (hpi) (Figure 1B). Also, the decrease in sialic acid level was detected as early as 1 hpi, suggesting that the virus entry into the cells may partially halt the production of sialic acid within an hour. Although the data were normalized to the non-infected control samples resulting in high variance, it was suggested that these differences were significant (Figure S2).

### Bioimaging of the expression levels of α-2,6-sialic acid on the membrane of the virus-infected cells using nanoprobes

**Figure 2:**
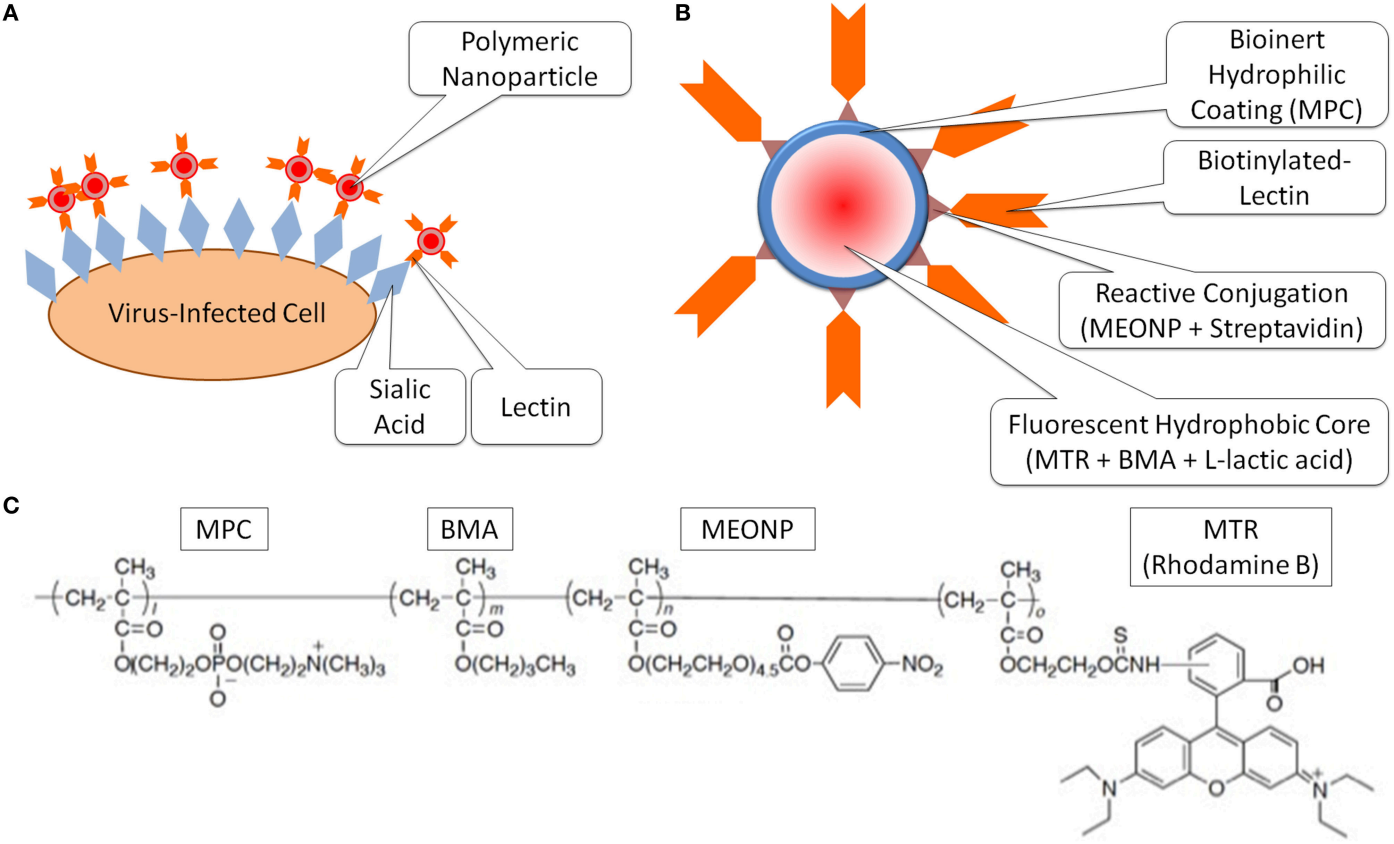
**The schematics of the experimental design (A), the design of the nanoprobe to detect sialic acids (B) and the chemical structures of the incorporated functional monomers (C)**.

**Figure 3:**
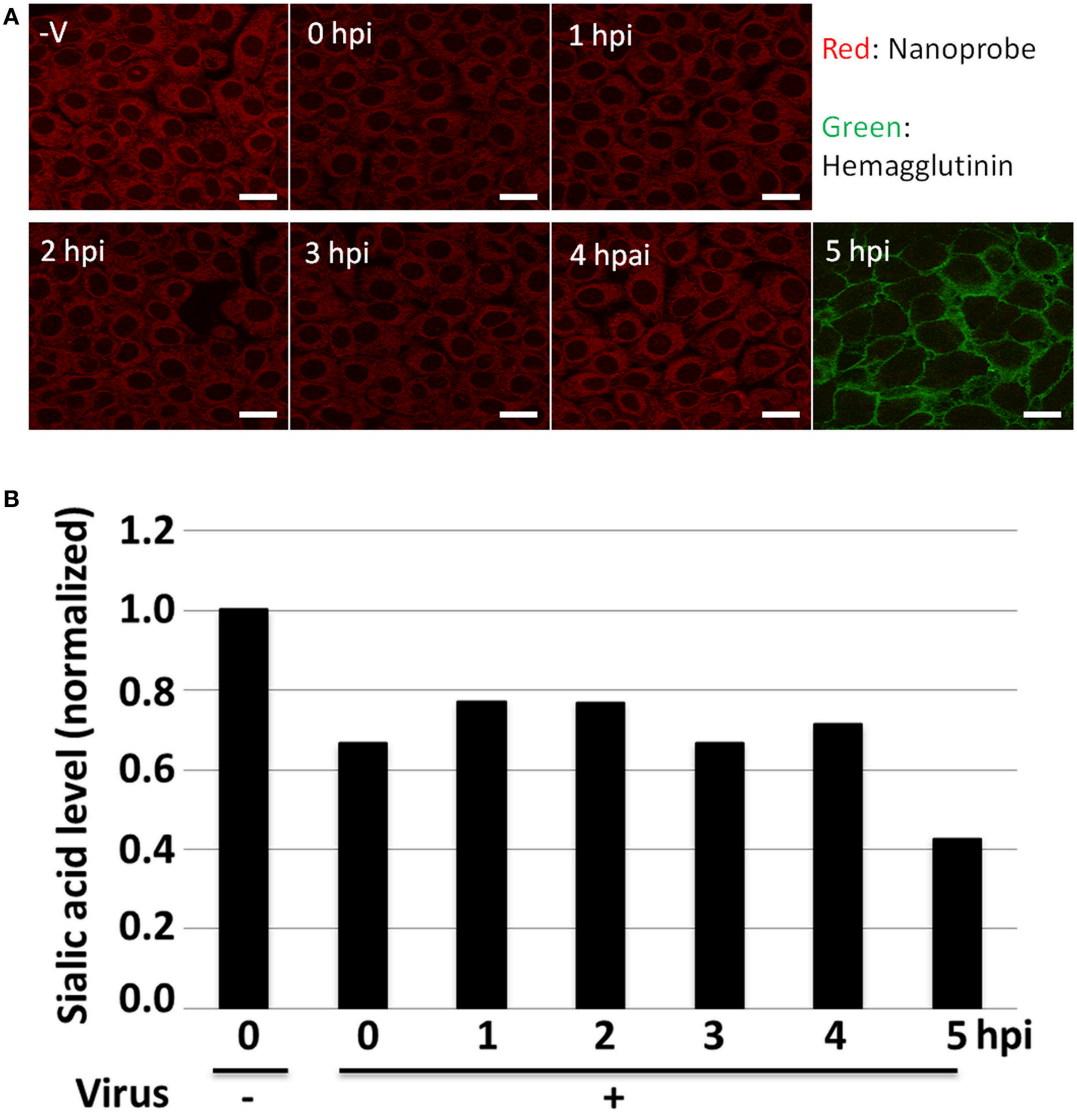
**The detection of membrane-bound α-2,6-sialic acids visualized using the nanoprobes**. **(A)** The fluorescence images of virus-infected H292 cells reacted with SNA-conjugated fluorescent polymeric nanoparticles [Red; viral infection time from 0 to 5 h post-infection (hpi)]. Controls without viral infection (–V) and the presence of HA (Green) after the viral infection are also shown. Scale Bar: 20 μm. **(B)** The normalized fluorescence intensities of virus-infected H292 cells reacted with SNA-conjugated fluorescent polymeric nanoparticles [viral infection time from 0 to 5 h post-infection (hpi)]. Result for the control without viral infection (–) is also shown and the data set was normalized to the control.

Next, the novel approach to detect the expression levels of the sialic acid on the virus-infected cells through bioimaging with the nanoprobes was tested (Figure 2). The specificity of these nanoprobes to α-2,6-sialic acid has already been confirmed in the previous study; when cells were pretreated with various sialidases that cleave sialic acids, the nanoprobes did not bind to the cells (Cho et al., 2014; Fujii et al., 2016). Furthermore, it was demonstrated that the lectins tethered to the nanoparticles have enhanced affinity for sialic acid compared to bare, fluorescently-tagged lectins (Figure S3), presumably due to the enhanced avidity of having multiple lectins on the nanoprobe surface. Thus, rapid and efficient analysis of sialic acid on the cell membrane was enabled, and the expression level of α-2,6-sialic acid on the cell membrane was analyzed during the viral infection time from 0 to 5 hpi. These nanoprobes bound the α-2,6-sialic acids and allowed the observations of the changes in the expression levels of the sialic acids by infection time (Figure 3A). The expression level of sialic acid was sharply decreased from immediately (0 h) after the infection, and followed a gradual decline thereafter (1~5 h). Also, HA expression on the cell was observed by immunostaining using FITC-conjugated anti-HA antibody (Figure 3A, 5 hpi), confirming the influenza virus infection.

To quantify the expression level of each sialic acid on the membrane of the virus-infected cells via the nanoprobes, the fluorescence intensities of 50 randomly-picked, representative cells were analyzed. The result showed the quantitative changes of the expression level of sialic acid against viral infection time (Figure 3B). For α-2,6-sialic acid, there was indeed a sharp decrease in fluorescence immediately after the infection and also at 5 h post-infection. Although the data were normalized to the non-infected control samples resulting in high variance, it was suggested that these differences were significant (Figure S4).

It is also important to note that nanoprobes are mostly taken up by the cells through endocytosis, as suggested by the fact that the decrease in temperature (to 4°C) causes a sharp decline in the uptake of nanoprobes (Figure S5), presumably due to the loss of fluidity of the membrane. The role of endocytosis was further confirmed using endocytosis inhibitors, such as nystatin and chlorpromazine, which suppressed the nanoprobe intensities to roughly 70 and 50% of the non-treated cells, respectively (data not shown). Thus, the observed nanoprobe fluorescence represent both the surface sialic acids on the outer cell membrane, as well as the endocytosed sialic acids, but not the newly synthesized sialic acids that are not membrane bound.

### Confirmation of the production of viral proteins in the cells through RT-PCR and immunostaining

To analyze the actual virus activity within the virus-infected cells and correlate it to the expression levels of the sialic acid, the influenza A virus were pre-incubated with cells for 1 h and the subsequent production of the viral proteins within the cells were analyzed. As before, the expression levels of sialic acid on the virus-infected cells were analyzed using lectin-conjugated polymeric nanoparticles. The production of the viral protein PB1, a crucial component of the viral RNA polymerase, was tracked at the level of transcription and translation. Thus, in parallel with the analysis of the expression levels of sialic acid on the incubated cells with viruses using lectin-conjugated polymeric nanoparticles, the analysis of viral protein production were performed by RT-PCR and immunostaining.

**Figure 4:**
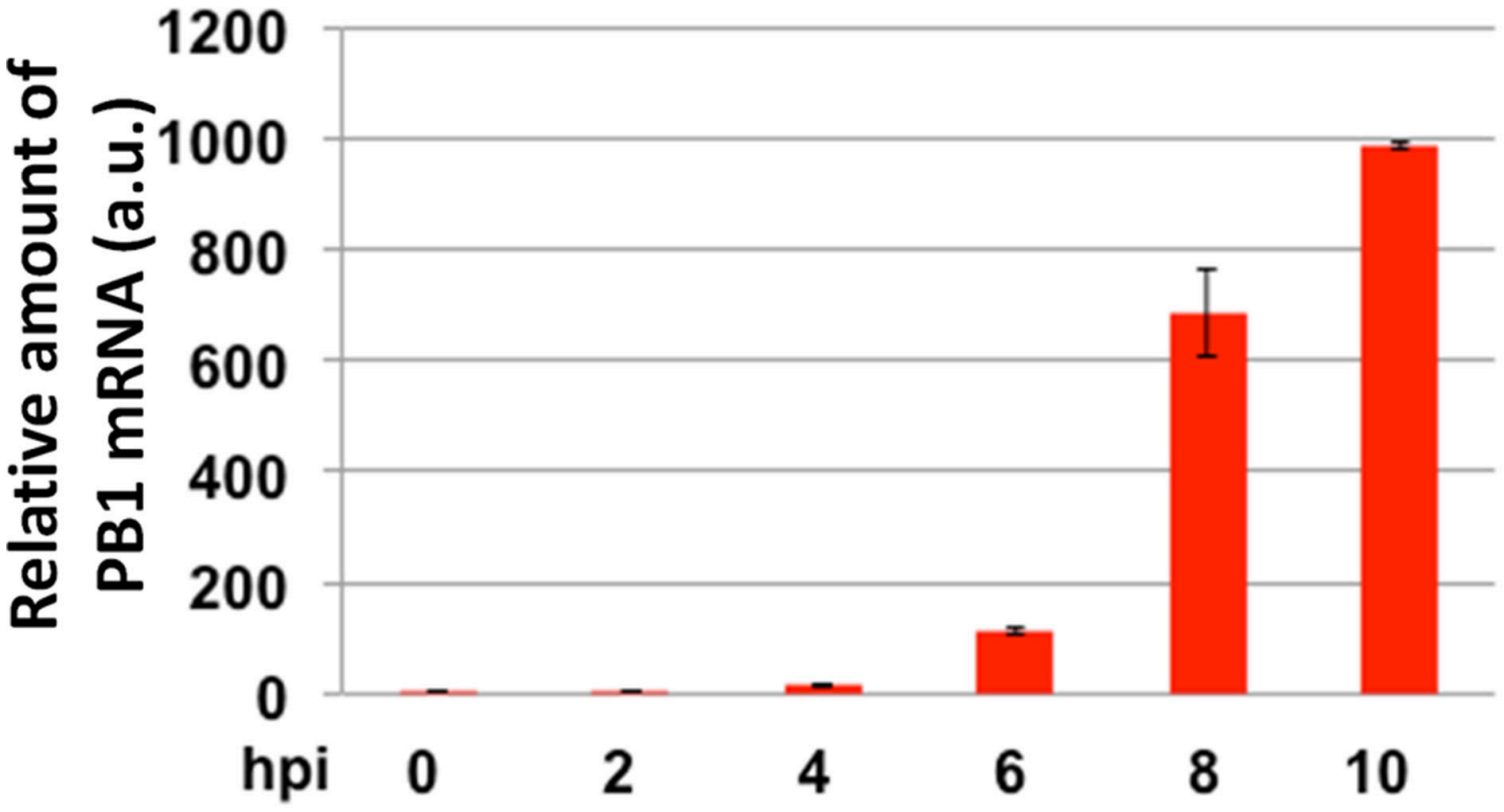
**The RT-PCR analysis of the amounts of mRNA coding for PB1 in cells with different hours post-infection (hpi)**.

First, RT-PCR for mRNA of PB1 was performed to confirm that the observation of sialic acid expression level obtained from the previous time-lapse data was due to the actual viral infection. From the RT-PCR data, the transcription of mRNA coding PB1 was analyzed against viral infection time (Figure 4). It was observed that the expression of mRNA of PB1 gene began to rise at past 5 h of viral infection and rapidly increased over time (Table S1). The time-course of PB1 mRNA production as observed through the RT-PCR experiment was consistent with the decrease in sialic acid expression levels observed in the previous experiments.

**Figure 5:**
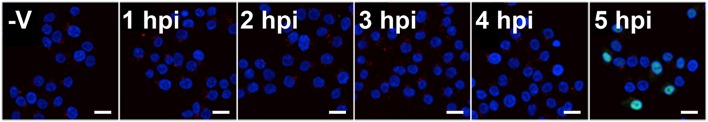
**The fluorescence images of the immunostaining of non-infected cells (–V) and virus-infected cells from 1 to 5 h post-infection (hpi) (Blue: nucleus; Red: nanoprobes, Green: PB1 viral protein)**. Scale Bar: 20 μm.

Furthermore, immunostaining was performed to monitor the production of PB1 in relations to the sialic acid expression levels of cells. The immunostaining was performed using anti-PB1 antibody and the fluorescent nanoparticles (Figure 5). The virus-infected cells were identified by the DAPI stain of the nucleus, while PB1 produced by the virus were tagged with the green fluorescence originating from the secondary antibody. The red signals from the nanoprobes seemed to be significantly reduced by the fixation process. Regardless, it was observed that the production of PB1 started at around 4 h post-infection, and stronger signals were obtained at 5 h post-infection (Figure S6). Again, the time-course of PB1 production as observed through the immunostaining experiment was consistent with the decrease in sialic acid expression levels observed in the previous experiments. Furthermore, the relative intensities of the signals showed a similar trend with the RT-PCR result of PB1 mRNA, where the production levels of PB1 was minute at 4 h and rose rapidly at 5 h.

## Discussions

All in all, the results demonstrate the accuracy and efficiency of the use of nanoprobes to monitor the changes in sialic acids during viral infections, which may serve as an indicator of viral infection progression. Because the change of expression level of sialic acid on virus-infected cells has not been previously demonstrated, it was confirmed by the detection of viral proteins using immunostaining and Western blotting in this study. However, it is important to note that the observation of the expression levels of sialic acid using the nanoprobe is much simpler and faster than immunostaining and Western blotting. This bioimaging method, which does not involve cell lysis protocol or electrophoresis, was performed in less than 1 h, and it offers the means to observe the changes in cellular properties *in situ*. Furthermore, it was found that the observation of the changes in the expression level of sialic acid can be observed from 1 h after viral infection, while the detection of PB1 can only be possible from roughly 5 h after a viral infection, suggesting that earlier detection of viral infection can be made possible using the nanoprobe platform.

Some findings in our studies warrant further research and investigation, such as the immediate decrease in the membrane-bound sialic acid levels after viral infection observed using the nanoprobes. To the best of our knowledge, there has not been any report on this immediate decrease in sialic acids during virus infections. However, there are various possibilities for the observed decline in the amount of membrane sialic acid immediately following virus infection. One possibility is that the fluidity of the cell membrane may be affected by virus infection, such that the endocytic process is inhibited, leading to less overall nanoprobe uptake of the cells. Another possibility is that the cell cycle progression is halted at G0/G1 phase during Influenza A virus infection, as shown in previous research (He et al., 2010), which may be hindering the production of sialic acids that generally occur at the end of G2 phase (Rosenberg and Einstein, 1972). Yet another possibility is that the process of virus binding itself may be depleting the available sialic acids on the cell membrane or withholding the endocytosed sialic acids, leading to the apparent decline in the amount of sialic acids on the cell membrane as quantified by the nanoprobe. Any of the above reasons or combinations thereof may be causing the initial decline in sialic acid levels on the cell membrane, but these possibilities still have to be further tested.

Furthermore, differences between the levels of sialic acid on the membrane of the cells (observed through the nanoprobe) and that of the entire cell (observed through the Western Blotting) were found. In particular, the sialic acid levels observed by the nanoprobe dropped immediately after virus infection, while the levels from the Dot Blotting dropped about 1 h post-infection. On the other hand, there was another decline of sialic acid levels at 4 h observed by Dot Blotting, while the nanoprobe detected this decline at 5 h. The former may be explained by some of the explanations listed above regarding endocytosis and receptor obstruction. The latter decline of the sialic acid will need to be further investigated, but it may be related to the diversion of metabolic pathways toward viral proteins instead of the sialic acids, which had a slightly delayed effect on the cell-surface sialic acids.

Lastly, the robustness and the generality of this nanoprobe system for various applications should be discussed. Recently, the mechanisms between different interactions between viruses and sialic acids and their structural correlations have become more apparent (Stencel-Baerenwald et al., 2014). Although the results here are that of one type of sialic acid and lectin combination, other forms of sialic acid, such as α-2,3-sialic acid that specifically interact with *Maackia amurensis II* lectin (MAL), should be tested in order to confirm some of the previous findings and gain further insight into the changes in sialic acid expressions upon viral infections. Furthermore, although the influenza virus infection occurs through the process of sialic acid recognition and the subsequent uptake of virus particles into the cells, other viruses often infect through different mechanisms. The nanoprobes use in this study, comprised of the lectin-tagged polymeric nanoparticles, contains lectins that recognize sialic acids, but they can also be conjugated with other molecules to capture different interactions and properties of the cell membrane surface, which will not only be useful for studying virus infections, but also to investigate various cellular functions as well.

## Conclusion

In this study, the behaviors of sialic acid on the influenza-virus-infected cells for viral infection time were analyzed using various methods. Immediate decline in the sialic acid levels were observed by the nanoprobe and Dot Blot analyses. After 4~5 h post viral infection, the amounts of sialic acid again decreased sharply, and this was consistent with the initiation of the viral protein production as confirmed by some of the traditional analytical methods. With the nanoprobe system, such analyses and the detection of the sialic acids relevant to virus-infected cells could be performed quickly, requiring only 15 min for the reaction with cells. In conclusion, the polymeric nanoparticle platform offers a rapid and accurate detection of sialic acids on the cell membrane that may find useful applications in the areas of disease control and clinical diagnosis in the future.

## Author contributions

JC, YM, AH, KK, and MT designed research; JC and YM performed research; JC and YM analyzed data; JC, YM, AH, KK, and MT wrote the paper.

### Conflict of interest statement

The authors declare that the research was conducted in the absence of any commercial or financial relationships that could be construed as a potential conflict of interest.
