# Supplementary material for: Analysis of the Changes in Expression Levels of Sialic Acid on Influenza-Virus-Infected Cells Using Lectin-Tagged Polymeric Nanoparticles
Source: Front Microbiol. 2016 Jul 21;7:1147. doi: 10.3389/fmicb.2016.01147 (PMC4954814; doi:10.3389/fmicb.2016.01147)
Supplement: Supplementary file 1 [file DataSheet1.pdf]

Supplementary Material

Analysis of the changes in expression levels of sialic acid on influenza-virus-infected cells using lectin-tagged polymeric nanoparticles

Jaebum Cho, Yukari Miyake, Ayae Honda, Keiichiro Kushiro, Madoka Takai*

Supplementary Figures

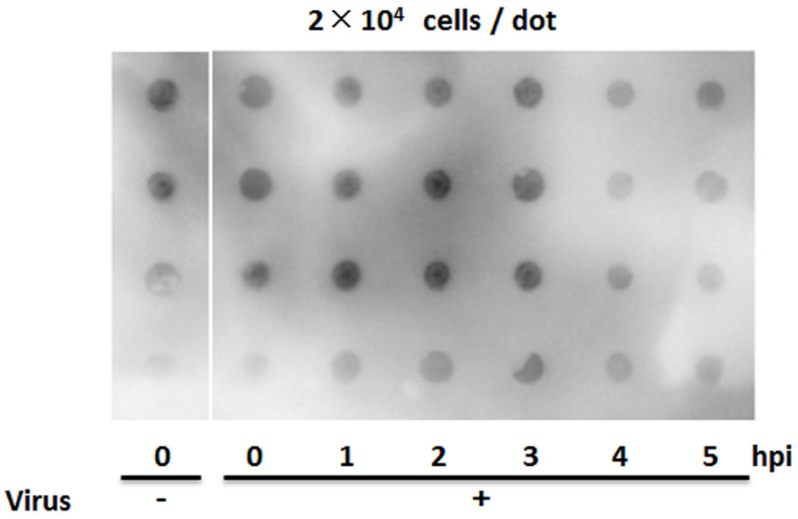

Figure S1. A representative dot blot assay with four duplicates for the different cell infection times, denoted as hours post-infection (hpi).

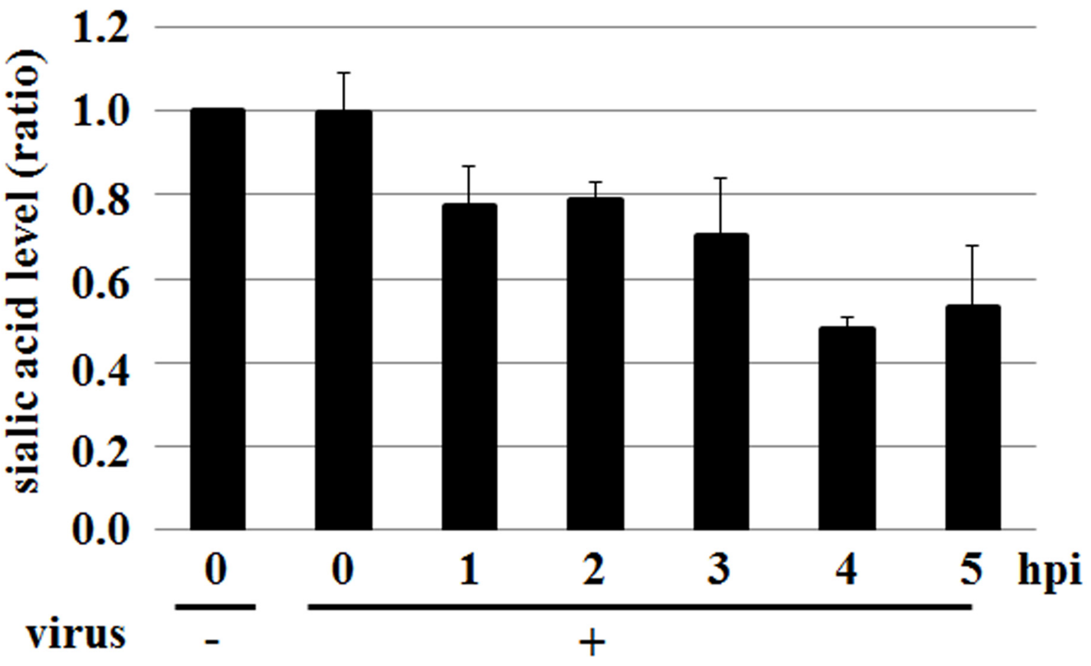

Figure S2. Versions of Figure 1B with error bars for reference.

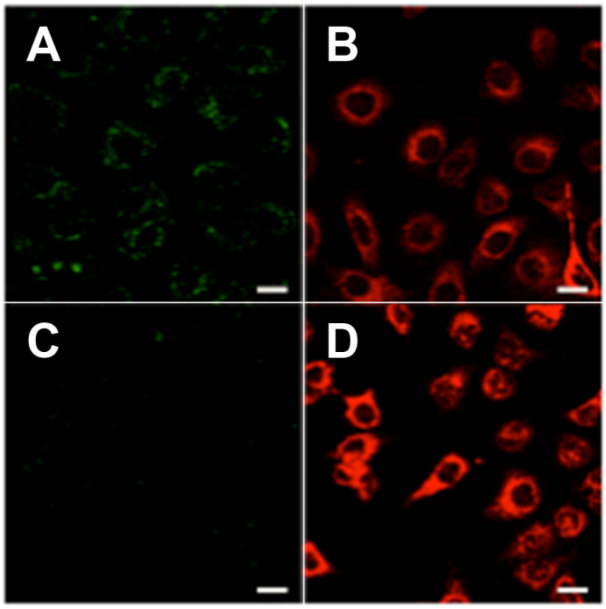

Figure S3. The fluorescence images of HeLa (A, B) and MCF-7 (C, D) reacted with SNA lectins conjugated with Alexa Fluor 488 (A, C) and SNA-lectin-conjugated fluorescent nanoparticles (B, D) after 2 hours of reaction. Scale Bar: 20 μm.

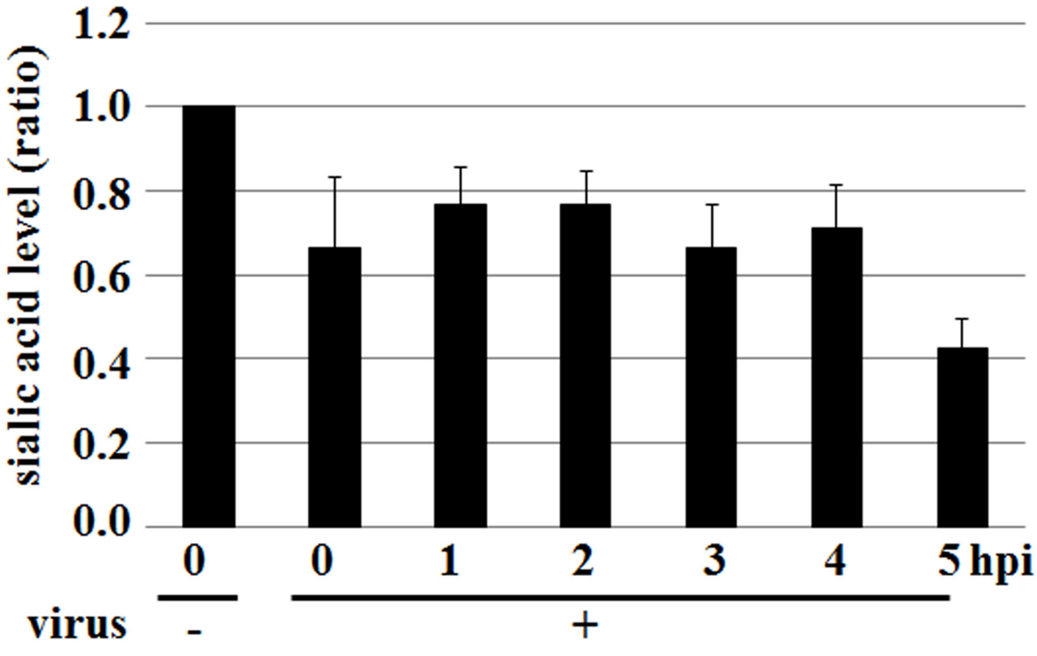

Figure S4. Versions of Figure 3B with error bars for reference.

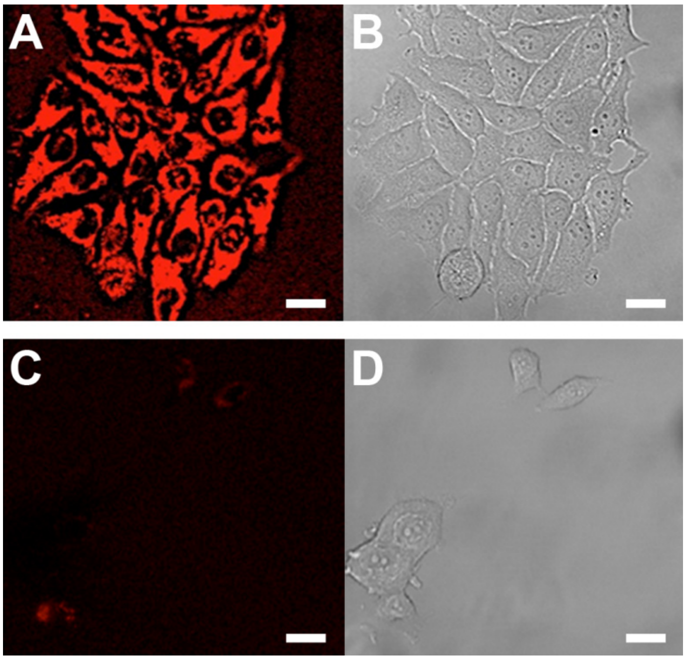

Figure S5. The fluorescence images (A, C) and bright-field images (B, D) of MCF-7 at 30 minutes post-incubation with nanoprobes at 37°C (A, B) and 4°C (C, D). Scale Bar: 20 μm.

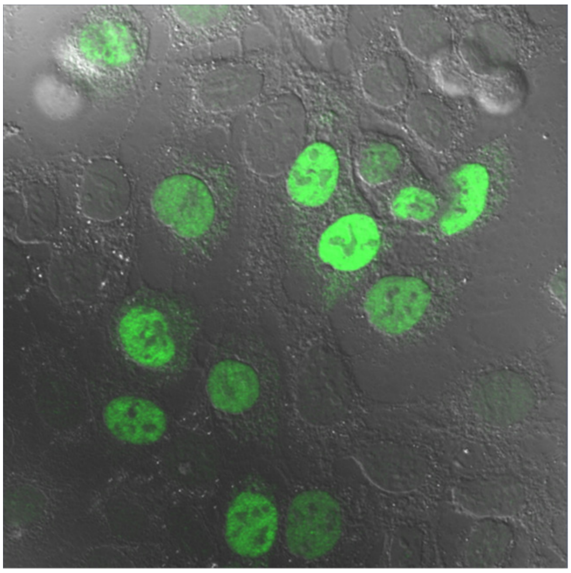

Figure S6. Alternative image of PB1 immunostaining detection at 5 hpi. (Green: PB1 viral protein)

Supplementary Table

Table S1. Expression level of RNA 2 (gene of PB1) in the early time points (0~5 hpi) was assayed using RT-PCR. –v represents the non-infected control samples, to which the statistical p values were compared. Ten samples were assayed.

hours post infection (hpi)	Cq. Value (p)
-v	Not detectable
0	Not detectable
1	37 (<0.03)
2	37 (<0.03)
3	31 (<0.01)
4	30 (<0.01)
5	28 (<0.01)
